# Measure of activity performance of the hand (MAP-Hand) questionnaire: linguistic validation, cultural adaptation and psychometric testing in people with rheumatoid arthritis in the UK

**DOI:** 10.1186/s12891-018-2177-5

**Published:** 2018-07-31

**Authors:** Yeliz Prior, Alan Tennant, Sarah Tyson, Ingvild Kjeken, Alison Hammond

**Affiliations:** 1 Centre for Health Sciences Research, University of Salford, Salford, UK; 2 Swiss Paraplegic Research, Nottwil, Switzerland; 3 Division of Nursing, Midwifery & Social Work, University of Manchester, Manchester, UK; 4 Department of Occupational Therapy, Prosthetics and Orthotics, Oslo and Akershus University College of Applied Sciences, Oslo, Norway

**Keywords:** PROMS, Patient reported outcome measures, Hand activity performance, Hand function, Hand pain, Psychometric testing, Rasch analysis, Validity, Reliability

## Abstract

**Background:**

Developed in the Norway, the Measure of Activity Performance of the Hand (MAP-Hand) assesses 18 activities performed using the hands. It was developed for people with rheumatoid arthritis (RA) using patient generated items, which are scored on a 0–3 scale and summarised into a total score range (0 to 54). This study reports the development and psychometric testing of the British English MAP-Hand in a UK population of people with RA.

**Methods:**

Recruitment took place in the National Health Service (NHS) through 17 Rheumatology outpatient clinics. Phase 1 (cross-cultural adaptation) involved: forward translation to British English; synthesis; expert panel review and cognitive debriefing interviews with people with RA. Phase 2 (psychometric testing) involved postal completion of the MAP-Hand, Health Assessment Questionnaire (HAQ), Upper Limb HAQ (ULHAQ), Short-Form 36 (SF-36_v2_) and Disabilities of the Arm Shoulder Hand (DASH) to measure internal consistency (Cronbach’s alpha); concurrent validity (Spearman’s correlations) and Minimal Detectable Difference (MDC^95^). The MAP-Hand was repeated three-weeks later to assess test-retest reliability (linear weighted kappa and Intra-Class Correlations (ICC (2,1)). Unidimensionality (internal construct validity) was assessed using (i) Confirmatory Factor Analysis (CFA) (ii) Mokken scaling and (iii) Rasch model. The RUMM2030 software was used, applying the Rasch partial credit model.

**Results:**

In Phase 1, 31 participants considered all items relevant. In Phase 2, 340 people completed Test-1 and 273 (80%) completed Test-2 questionnaires. Internal consistency was excellent (α = 0.96). Test-retest reliability was good (ICC (2,1) = 0.96 (95% CI 0.94, 0.97)). The MAP-Hand correlated strongly with HAQ20 (r_s_ = .88), ULHAQ (r_s_ = .91), SF-36_v2_ Physical Functioning (PF) Score (r_s_ = −.80) and DASH (r_s_ = .93), indicating strong concurrent validity. CFA failed to support unidimensionality (Chi-Square 236.0 (df 120; *p* < 0.001)). However, Mokken scaling suggested a probabilistic ordering. There was differential item functioning (DIF) for gender. Four testlets were formed, resulting in much improved fit and unidimensionality. Following this, testlets were further merged in pairs where opposite bias existed. This resulted in perfect fit to the model.

**Conclusions:**

The British English version of the MAP-Hand has good validity and reliability in people with RA and can be used in both research and clinical practice.

**Electronic supplementary material:**

The online version of this article (10.1186/s12891-018-2177-5) contains supplementary material, which is available to authorized users.

## Background

Rheumatoid arthritis (RA) is a chronic autoimmune disorder affecting joints and surrounding tissues [1]. Most commonly, RA results in swollen, hot and painful joints and generalised stiffness, which worsens with rest. The hands and wrists are the most commonly affected joints. Typically, metacarpophalangeal (MCP) and proximal interphalangeal (PIP) joints become swollen and painful. As a result, people struggle with daily activities requiring gripping, pinching and carrying. If unresolved, these difficulties may lead to activity limitation, participation restriction and loss of independence in later life [2]. Therefore, early recognition and rehabilitation of hand pain and problems may help to improve people with RA’s future health and quality of life.

The National Institute for Clinical Excellence (NICE) Guidelines for the Management of Adults with RA [3] recommend that patients should have access to specialist occupational therapy if they have difficulties with hand function. To maintain or improve these abilities, occupational therapists need to effectively identify individual’s difficulties and evaluate therapy outcomes. To this end, valid, reliable patient-informed Patient Reported Outcome Measures (PROMs) that are relevant to the interventions rheumatology occupational therapists provide are necessary, but there is only one appropriate measure is currently validated for use in the UK [4]. Several self-reported measures of hand function are available for use in RA [5–8] however these did not involve patients in their development [9]. It is increasingly recognised that patients should inform the development of such measurement tools [10] to ensure that issues most relevant and important to them are included.

The Measure of Activity Performance of the Hand (MAP-Hand) questionnaire is an 18-item PROM of hand activity performance, which was developed and rigorously tested in Norway. It has good evidence of reliability and validity in Norwegian people with RA (*n* = 134) [9]. To ensure items were representative of normal hand function, items were matched to the eight main handgrips using the Sollerman handgrip classification [11]. Rasch analysis was used to finalise the 18-itemstructure representing a range of item difficulty. The scale is unidimensional and has a high person separation index of 0.93. Test-retest reliability is good (ICC = 0.94) although only conducted with 34 people. The MAP-Hand significantly correlated with the AIMS_2_ hand and finger function (*r* = 0.78) and arm scales (*r* = 0.66) [9]. Following testing the MAP-Hand was translated into North American English in accordance with the recommended translation procedures for scale development [9]. The MAP-Hand developers recommended further testing in different countries to establish its psychometric properties and cultural validity.

Linguistic translation of self-administered questionnaires for use in different cultural contexts is insufficient [12, 13]. Researchers must also ensure cross-cultural adaptation to establish items are relevant and understandable to the population of interest, and whether additional items need including to avoid systematic bias [12]. Once adapted, further psychometric testing is required to ensure content validity and reliability is retained across different cultures [12, 13]. Beaton et al. [12] published guidelines for cross-cultural adaptation of self-report measures to standardise this process. A decade following this, Consensus-based Standards for the selection of health Measurement Instruments (COSMIN) checklist was developed to evaluate the methodological quality of the studies reporting measurement qualities [14, 15]. More recently COSMIN methodology for evaluating the content validity of patient-reported outcome measures were proposed as a rating system to summarise the evidence of a PROM’s content validity [16]. This is deemed to be more detailed, standardised, and transparent than earlier published guidelines, including the previous COSMIN standards [16].

The overall aim of this study was to develop a British English version of the MAP-Hand following the recommended linguistic and cultural adaptation guidelines and test its psychometric properties (internal consistency, construct and concurrent validity, test-retest reliability and minimal detectable difference) using both the classical testing theory and item response theory in a UK population of people with RA.

## Method

### Study setting

Participants were recruited from rheumatology and occupational therapy departments in 17 National Health Service (NHS) Hospitals across the UK.

### Eligibility criteria

Within a test-retest design it is important to ensure participants’ disease status is clinically stable at two time points to avoid risk of bias [14]. Therefore a recent change in medication may result in changes of the participant’s hand function and/or physical and mental health status. Patients were screened at the rheumatology outpatient clinics by research nurses and occupational therapists using an eligibility checklist and excluded if they are about to or recently started (during the last 3-months) or increased dose of a biologic or disease modifying anti-rheumatic drugs (DMARDs), low dose oral steroids or received an intra-muscular steroid injection, had cognitive impairment affecting ability to understand and complete the study questionnaire; had another health condition(s) which is moderately to severely affecting their ability to participate in activities and/or hand function; had mental health problem(s) or terminal illness and it was inappropriate to request participation; or were unable to provide informed, written consent. However, people with Fibromyalgia, Osteoarthritis or other conditions that is secondary to RA (e.g. heart disease) were not excluded from the study. Those who were aged 18 years and above: able to read, write and understand English, and diagnosed with RA by a rheumatology consultant were included in the study providing written informed consent was obtained.

### Procedures

#### Phase 1: Cross-cultural adaptation

The linguistic validation and cross-cultural adaptation guidelines were followed [12–14]. As a North American English version of the MAP-Hand was already available from the Norwegian developers, backward translation was not required (Additional file 1). Two native British English speakers forward translated the MAP-Hand; one of whom was unfamiliar with health outcome measures and not involved in health care; and the other was a rheumatology health professional. Translators synthesised translations to resolve discrepancies and an Expert Panel reviewed translations to agree a pre-final British English MAP-Hand. The Expert Panel included health professionals (occupational therapists and a physiotherapist), native English speakers, a methodologist and a layperson with RA. The panel reviewed the MAP-Hand for semantic (i.e. do words mean the same thing?), idiomatic (e.g. presence of colloquialism or idioms), experiential and conceptual equivalence.

### Cognitive de-briefing interviews

A purposive sample of participants with wide range of demographic characteristics and health status were identified from the past participants of the EDAQ [Evaluation of Daily Activity Questionnaire] study [17, 18] within five rheumatology outpatient departments in the North West of England. They were mailed an invitation letter, participant information sheet, reply form and a FREEPOST envelope. Upon receipt of a positive reply, they were telephoned by an occupational therapist to go through the eligibility checklist, explain what the study involves and provide an opportunity to answer any questions they may have prior to deciding to take part. Consenting participants were booked in to partake in a cognitive de-briefing interview and mailed the Phase 1 questionnaire booklet to complete at home, in their own time no sooner than 1 week prior to the arranged telephone or face-to-face interview. These semi-structured interviews were aimed to ascertain whether the participant found the MAP-Hand items relevant, understandable and comprehensive (i.e. did they adequately reflect the most common daily activity difficulties experienced when using their hands and whether any additional items should be included). Such interviews are recommended during PROM development to ensure participants’ understanding of their content matches the intended use [12]. The interviewer used a five-point rating scale to assess the relevancy and ease of comprehension of each item in the MAP-Hand (relevancy was measured as 1 = not relevant to 5 = very relevant; and comprehension was measured as 1 = very easy to understand to 5 = very difficult to understand). Interviews were audio-recorded, transcribed and analysed to identify the need for recommended changes and/or the inclusion of new items. A summary of the findings was reviewed by the Expert Panel to decide whether further changes were required. Following this, a detailed report of the linguistic validation and the cross-cultural adaptation process taken place and the finalised British English MAP-Hand were submitted to the Norwegian developers for review and approval.

#### Phase 2: Psychometric testing

It is highly recommended that culturally adapted questionnaires should be further tested following the cross-cultural validation process to ensure the new version demonstrated the psychometric properties needed for the intended application [12]. Therefore Phase 2 consisted of psychometric testing of the British English MAP-Hand Questionnaire.

### Participants

During Phase 2, participants were recruited from rheumatology outpatient clinics within 17 NHS hospitals across the UK. These included both rural and urban populations and a wide mix of socio-demographics.

### Data collection

Participants were mailed a Test 1 questionnaire booklet, which included demographic and health data (e.g. age, gender, marital, educational and employment status, disease duration, medication) and following outcome measures: the (i) Health Assessment Questionnaire (HAQ) which includes ability to perform 20 daily activities rated on a 0–3 scale (0 = not at all difficult; 3 = unable to do) [19] and Upper Limb HAQ (ULHAQ)[7 Upper Limb HAQ items] [20]; the Medical Outcomes Survey 36 item Short-Form 36 (SF-36_v2_) from which sub-scale of Physical Function was selected [21, 22]; British English Disabilities of the Arm Shoulder Hand (DASH) which consists of 30 items, measured using five-point Likert scales, 21 daily activity ability items, five symptom items, three participation items, and one of self-image [4]; and Symptom Numeric Rating Scales (NRS) from the EDAQ Part 1, rating hand and wrist pain and arthritis severity on a 10-point scale [17, 18].

Participants were mailed a repeat [Test 2] questionnaire booklet two to 3 weeks later to complete at home to conduct the test-retest reliability of the British English MAP-Hand. Test 2 questionnaire booklet only included brief items on basic demographics (i.e. date of birth, gender and postcode); two single items about current health status and functioning (i.e. “Considering all the ways that your condition affects you, how have you been over the past month?” and “Overall, how much your arthritis is troubling you now compared to when you last completed this questionnaire a few weeks ago?”) and the British English MAP-Hand for repeat testing (Additional file 2).

### Statistical analysis

#### Sample size

The sample size calculation suggested that, for Rasch analysis a sample size of 243 will give 99% confidence of the person estimate being within ±0.5 logits, irrespective of whether or not the scale is well targeted to the patients [23]. A minimum of 79 sets of repeated responses were required to demonstrate that a test-retest correlation of 0.7 differs from a background correlation (constant) of 0.45, with 90% power and 99% significance.

#### Unidimensionality

The MAP-hand is reported to be a unidimensional extant scale [9]. As such, confirmation of its structure from a classical test perspective would follow from a Confirmatory Factor Analysis (CFA), where a priori there is evidence that the item set constitutes one factor [24]. Although the Rasch model assumes unidimensionality, and this can be tested post-hoc, it can still be informative to examine the scale through a CFA, particularly as Mokken scaling also has this assumption. Following Kline, fit is determined by a non-significant chi square statistic [25]. Approximate (or ancillary) fit statistics include the Root Mean Square Error of Approximation (RMSEA) where a value less than 0.06 would be appropriate, the Comparative Fit Index (CFI), a comparison of final model and baseline model and the Tucker Lewis Index (TLI), another incremental fit Index which adds penalties for increasing the parameters. Both indices would suggest good fit with values above 0.95. Thus in the present study the item set is fit to a CFA model in Mplus using a polychoric correlation matrix [26].

### Mokken scaling

The Mokken scale is a non-parametric probabilistic model that utilises Loevinger’s H coefficient to determine the ‘scalability’ of a set of items. ‘H’ is a measure of the degree to which the score is able to discriminate between persons in the given sample [27]. It has been argued that Mokken scaling is a natural starting point for item analysis, and it is used here in that context, to identify if any items from the MAP-Hand display a level of discrimination inconsistent with the expectations of the Rasch model, as represented by low values (< 0.3) of H. In the present study Mokken scaling is examined through the *msp* procedure in STATA 13 [28].

### Construct validity

The Rasch model is widely applied to PROMs to ascertain if a quantitative structure is present for the domain(s) measured [29]. A practical realisation of additive conjoint measurement, where data are shown to meet the model expectations, it allows the transformation of ordinal data into an interval level latent estimate [30–34]. The model expectations are associated with a series of assumptions, or requirements, including the stochastic ordering of items (or fit), unidimensionality and local independence [35]. Fit is evaluated by several fit statistics, including chi-square statistics for items and in total (which should be non-significant, Bonferroni adjusted), standardised item and person residuals (within a range ± 2.5), and summary residuals with a mean of zero and standard deviation of one where data have perfect fit to the model. Local response dependency can be examined through the residual correlation matrix [36]. When the local independence assumption is violated, items can be grouped into ‘testlets’ which absorb the dependency [37]. When data are made into testlets, this delivers a bi-factor solution for the latent estimate, where any unique non-error variance is discarded [38].

A post-hoc test of unidimensionality was also undertaken following the approach described by Smith [39]. Finally, within the Rasch model framework, emphasis is placed upon the invariance of comparisons between groups, such that *at the same level of the trait* being measured (e.g. hand function), the probability of response to an item should be equal across groups, otherwise Differential Item Functioning (DIF) is present and will require adjustment [40, 41]. Consequently the process of fitting data to the Rasch model, widely referred to as Rasch analysis, consists of a series of tests related to the assumptions of the model, and adjustments to accommodate deviations from those expectations. This process, in relation to the measurement of health outcomes, is described in detail elsewhere [42].

In the current application, emphasis is placed upon fit to the model expectations, and invariance by contextual group, in this case by age, gender, employment and marital status, duration of disease and magnitude of disability as expressed by the HAQ. The analysis used the RUMM2030 software utilising the partial credit parameterisation of the Rasch model [43, 44].

### Concurrent validity

The MAP-Hand scores were compared with comparative health measures, specifically the HAQ-20 and ULHAQ [19]; SF-36_v2_ (Physical Functioning Score Norm-Based; General Health and Physical Component) [21, 22]; British English DASH [4]; numeric rating scales of hand and wrist pain (i.e. pain in the hand and wrist past week) and arthritis severity (i.e. effect of arthritis in the past month; pain when resting; pain when moving) [17]. Concurrent validity was measured using Spearman’s correlations between the MAP-Hand and these comparative health measures.

Reliability.

Internal consistency was measured using Cronbach’s Alpha (α) and the Person Separation Index (PSI) which, should the data have a normal distribution, is equivalent to Cronbach’s Alpha [45].

### Test-retest reliability

Test-retest reliability of the MAP-Hand was assessed using linear weighted kappas from Test-1 and Test-2 items and at scale level using Rasch transformed estimates and Intra Class Correlations (ICC) (2,1). Analyses were conducted using IBM SPSS Statistics v20 and MedCalc Statistical Software.

Measurement Error.

Measurement error was assessed by transforming the MAP-Hand scores into logits and linearly transforming them to produce an interval-scale. Following this, Standard Error of Measurement (SEM) and the minimal detectable change (MDC_95_) score were calculated [46, 47]. If ≥15% of responders achieved either the lowest or highest score, floor and ceiling effects were considered to be present [48, 49].

## Results

### Phase-1: Cross-cultural adaptation

Cognitive debriefing interviews were conducted with 31 participants. Participants’ socio-demographic and health characteristics are detailed in Table 1.

**Table 1:** MAP-Hand Study Participant Characteristics (*n* = 340)

Participant Characteristics	Cognitive debriefing [Phase-1] Participants (*n* = 31)	Psychometric testing [Phase-2] Participants (*n* = 340)
Age:(Mean (SD)	63.42 (12.04)	61.96 (12.09)
Gender (M:F)	5:26	89:251
Condition duration (years) (Mean (SD):	15.71 (12.61)	14.44 (11.73)
Marital status: *n* (%):		
Married/living with partner	23 (74%)	241 (71%)
Living status: *n* (%)		
Family/significant other	24 (77%)	245 (72%)
Children living at home	4 (13%)	36 (11%)
Employment status:		
Paid employment	3 (10%)	108 (32%)
Retired	22 (71%)	204 (60%)
Other	6 (19%)	28 (8%)
Education level (ISCED):		
Secondary education only	19 (61%)	182 (54%)
Current medication:		
Not on DMARDs	2 (6%)	34 (10%)
Monotherapy	10 (32%)	91 (27%)
Combination therapy	10 (32%)	190 (56%)
Biologic drugs	9 (29%)	25 (7%)

Overall, the interviews showed that the MAP-Hand items were both understandable and relevant, and take 2 minutes to complete. Specific items that were highlighted as potentially problematic were:

(i) Item 10 (slicing bread using a knife); as participants often bought sliced bread, this item was not applicable to most (*n* = 24). However, most responded to the item by recalling the last time they sliced bread, such as baguettes, as it is instructed.
(ii) Item 16 (type on a computer) was not applicable to some participants (*n* = 7) as they didn’t use computers. As the ‘not applicable’ option is not available in the response options, these participants either left this item blank or guessed their ability based on other activities require similar input (e.g. using a mobile phone to text) to answer.

Nevertheless, all items were deemed to be relevant by the participants and expert panel and therefore retained in the British English MAP-Hand. Cultural adaptations included making small changes in the wording of three items i.e. the item 7 was reworded as “opening screw top bottles” instead of “opening bottle screw tops”; the item 8 was reworded as “opening cans (any type)” instead of “opening hermetic cans” and the item 12 was reworded as “stirring food in a pan” instead of “stirring food in a pot” (Additional file 2).

### Phase 2: Psychometric testing

**Fig. 1:**
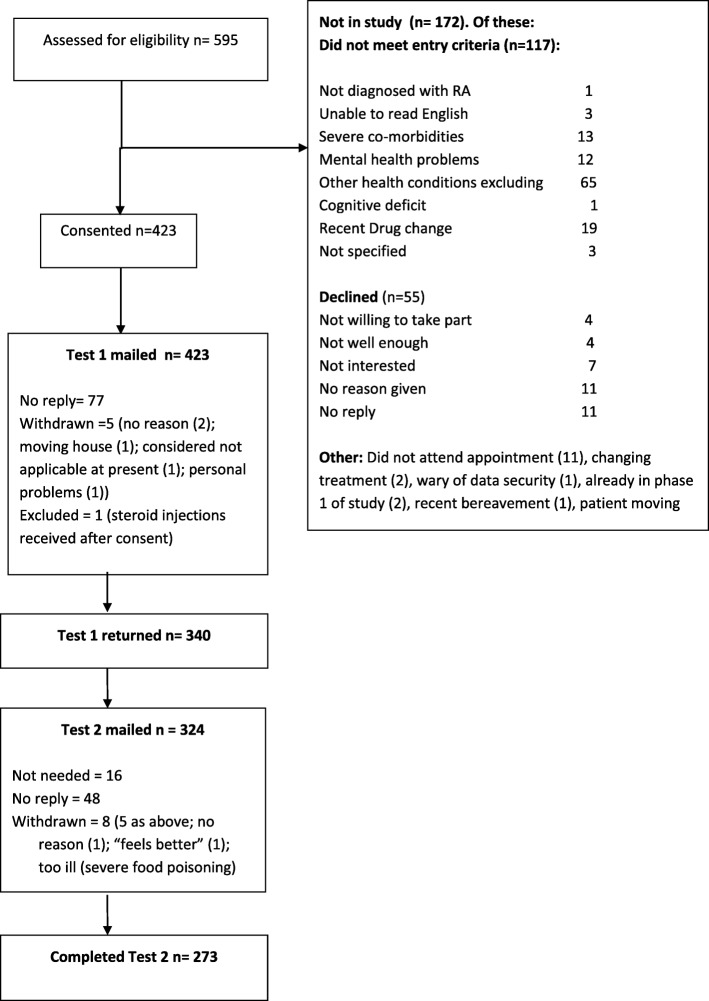
MAPHAND Recruitment & Study Progress Flow Diagram (Phase 2)

In Phase 2, 340 participants completed the Test 1 questionnaire and re-test was completed by the 80% (*n* = 273) of the responders. The recruitment progress is summarised in Fig. 1 and the participants’ socio-demographic and health characteristics are detailed in Table 1.

### Construct validity (Mokken and Rasch models)

A CFA failed to support the unidimensional structure of the 18-item set of the British English MAP-Hand (Chi-Square 236.0 (df 120; *p* < 0.001). RMSEA was 0.53 (90%CI:0.43–0.63); CFI 0.995; TLI 0.994. Mokken scaling suggested that all 18 items showed a probabilistic ordering with a moderate scaling level of 0.61. Initial fit of the 18 items of the MAP-Hand to the Rasch model showed some misfit to the model (Table 2, Analysis 1), and a significant breach of the unidimensionality assumption. DIF was largely absent across all contextual factors, but was present for gender for the item 3 ‘ tying shoelaces’, the item 7 ‘opening screw top bottles’ and the item 18 ‘carrying heavy objects’. At any level of hand function, males were more likely to score higher (worse) than females for tying shoelaces, and females were more likely to score higher than males with opening screw top bottles. Reliability was high, but possibly inflated by the local dependency.

**Table 2:** Rasch Analysis of the MAP-HAND (*n* = 340)

	Description	Chi- Square*	Df	P	Residual Item		Residual Person		Person Separation Index [PSI] Reliability	% tests > 5%	95%CI	*N*
					Mean	SD	Mean	SD				
1	MAP-HAND 18	113.17	72	0.001	−0.4073	1.4939	−0.2936	1.1725	0.95	13.4	11.0–15.7	340
2	Four testlet version	19.81	16	0.229	−0.197	2.6078	−0.4561	1.0624	0.92	5.36	3.0–7.8	340
3	Two testlet version	3.0	8	0.934	0.039	1.1792	−0.5832	0.8457	0.92	4.35	1.9–6.8	340
	Ideal Values			> 0.05*		< 1.4		< 1.4	> 0.70	< 5.0	LCI < 5.0	

*Bonferroni Adjusted

**Fig. 2:**
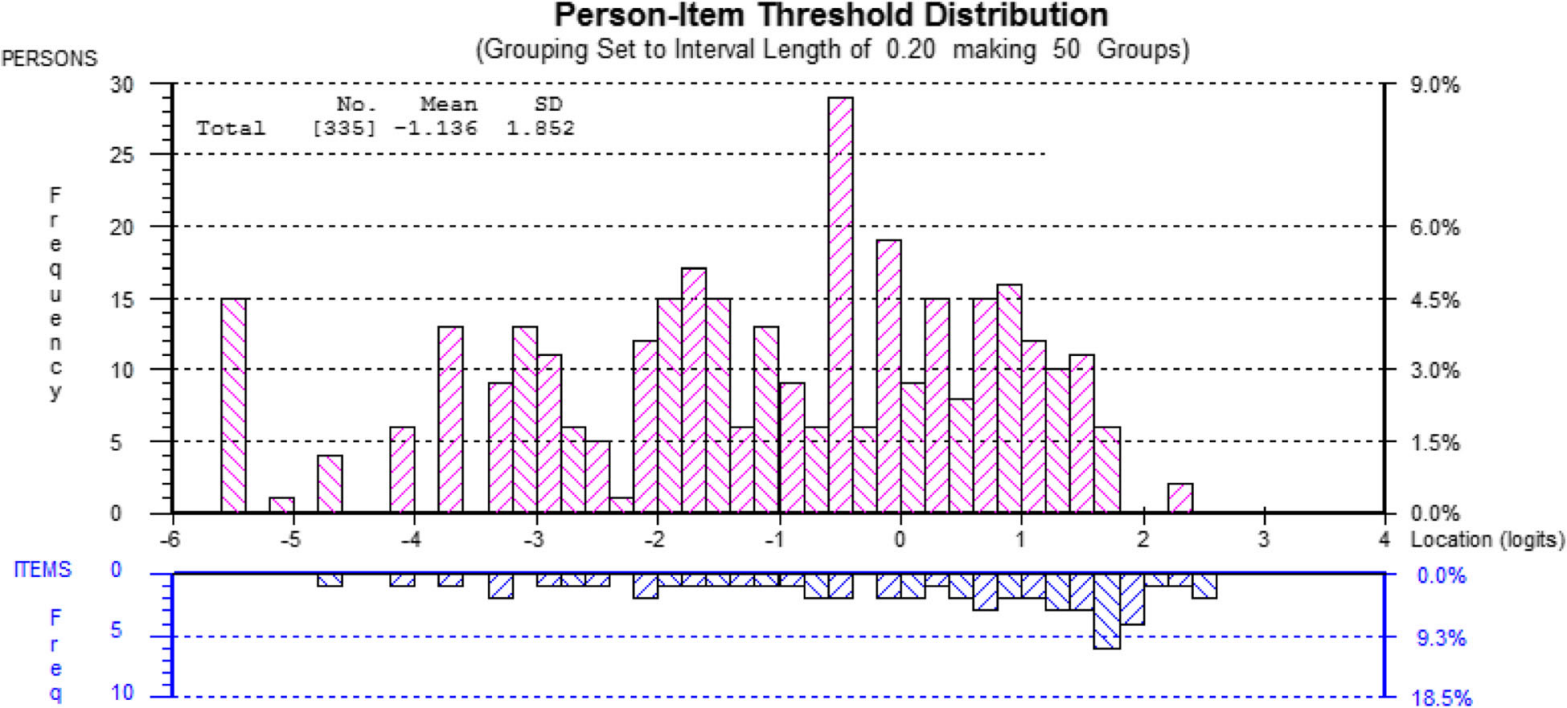
Distribution of persons-item threshold

Clusters of locally dependent items could be observed. For example, button, socks and laces, any form of opening jars or cans and carrying bags or heavy objects. Consequently four testlets were formed from the item set and the data refitted to the model. Here fit was much improved and the unidimensionality assumption held (Analysis 2). The average latent correlation between the four testlets was 0.91, and the amount of common non-error variance in the latent estimate was 0.97, meaning that just 3% of the non-error variance was discarded. This suggests the earlier breach of the unidimensionality assumption was caused by clusters of locally dependent items. Nevertheless, some gender DIF persisted. As earlier it was noted that some items favoured males, and others favoured females, the testlets were further merged in to pairs where opposite bias existed. This resulted in perfect fit to the model, and no DIF (Analysis 3). The scale and patients were slightly off-target in that the latter were more able (less difficulties) than the average of the scale (Fig. 2). However, the floor effect was minimal (5.6%). Table 3 provides the ordinal raw score- interval scale transformation.

**Table 3:** Transformation of Raw score to Interval metric

0	0.0
1	4.9
2	8.4
3	10.9
4	12.9
5	14.6
6	16.1
7	17.5
8	18.8
9	20.1
10	21.4
11	22.6
12	23.7
13	24.9
14	26.0
15	27.0
16	28.0
17	29.0
18	29.9
19	30.8
20	31.7
21	32.5
22	33.3
23	34.1
24	34.8
25	35.5
26	36.2
27	36.8
28	37.4
29	38.0
30	38.6
31	39.1
32	39.6
33	40.1
34	40.6
35	41.0
36	41.5
37	41.9
38	42.2
39	42.6
40	43.0
41	43.3
42	43.6
43	44.0
44	44.3
45	44.7
46	45.1
47	45.5
48	46.0
49	46.6
50	47.3
51	48.1
52	49.3
53	51.1

54	54.0

**Fig. 3:**
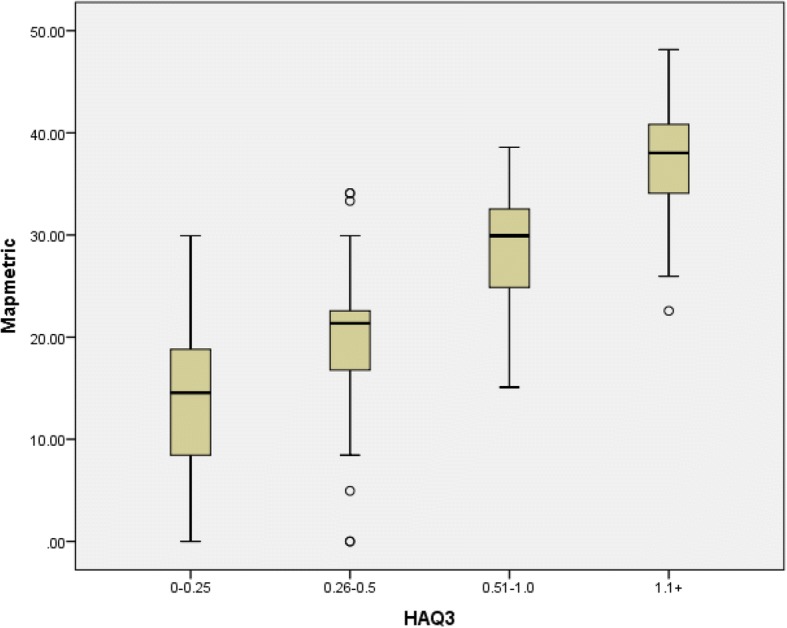
Boxplot of MAP-hand Metric for HAQ groups (*n* = 340)

A significant gradient of the transformed metric is seen across groups of functional limitation as defined by the HAQ (Table 4, Fig. 3) (ANOVA F = 217.1; *p* < 0.001). Females also showed more limitations in hand function than males (t-test; *t* = 3.1; *p* = 0.002). Duration of disease also showed a significant difference, mainly due to the group with duration over 21 years (ANOVA post-hoc tests). There was no significant difference by age group (ANOVA F = 0.254; p 0.851).

**Table 4:** MAP-hand metric across levels of the HAQ

HAQ	MAP-HAND
0–0.25	13.49
0.26–0.5	19.75
0.51–1.0	28.72
1.1+	37.28

### Concurrent validity

MAP-HAND correlated strongly with HAQ20 (r_s_ = .88), ULHAQ (r_s_ = .91), SF-36v2 (PF) Score (r_s_ = −.80) and DASH (r_s_ = .93), indicating strong concurrent validity (Table 5).

**Table 5:** Concurrent validity of the British English MAP-Hand with quality of life measures

	HAQ20	Hand HAQ [ULHAQ]	DASH	SF36v2 Physical Function
MAP-Hand	0.88**	0.91**	0.93**	−0.80**

Key: Spearman’s correlations; ** *p* <0.001

### Internal consistency (reliability)

The Map-Hand showed a high Person Separation Index reliability (PSI), even after adjustment for local dependency (PSI range: 0.95–0.92). Reliability measured by Cronbach Alpha (α) was also excellent (α = 0.96).

### Test-retest reliability

Test-retest reliability was good: at item-level linear weighted kappa scores were good (range 0.61–0.75); at scale level, the ICC (2,1) score was 0.96 (95% CI 0.94, 0.97).

### Measurement error

The SEM was 1.44 and the MDC_95_ score was 3.99. There was no floor or ceiling effects present.

### Summary of the results

The MAP-Hand questionnaire was linguistically validated and culturally adapted using the recommended guidelines [12–14] in a UK population of adults aged ≥18 years with RA [50]. The British English version of the MAP-Hand retained all original 18 items, with some changes to the phrasing of these and the instructions provided to make it easily understandable by British English speakers. The British English MAP-Hand was then psychometrically tested using both classical test theory and item response theory to provide quantitative assessments of the validity and reliability of this PROM in a UK population of adults with RA. The results of the analyses support the validity and the reliability of the British English Map-Hand [50, 51]. The raw score is a sufficient statistic for hand function, and an interval scale metric is provided on Table 3 [51].

## Discussion

The British English MAP-Hand is a brief, valid and reliable measure of hand activity performance, which can be completed in an average of 2 minutes by people with RA. Due to its ease of use and precision it is an ideal questionnaire to utilise in busy clinical environments, such as the NHS outpatient rheumatology and hand therapy clinics. As a psychometrically robust measure, it can be used to evaluate clinical outcomes, for research purposes, and to describe the patterns and extent of hand activity performance in people with RA in the UK.

### Implications for clinical and research practice

The British English MAP-Hand correlated highly with the HAQ20 and ULHAQ [4]. Although these outcomes are measuring similar constructs, the MAP-Hand was developed using patient generated items and this is reflected in the way in which the functional limitation is defined and scores are calculated. For instance, the hand activity performance is measured in the MAP-Hand at a person-level functioning (activity) [52] with or without the use of aids and adaptations (i.e. gadgets). This means that the MAP-Hand functional limitation score does not increase when the responder appraise their ability to do an activity such as “Writing by hand” (item 15) as “No difficulty” due to their use of pen grip to reduce pain and ease function. People with RA are increasingly encouraged to self-manage their condition, which includes adherence to joint protection advice, that may require behavioural modifications and the use of aids and environmental adaptations [53]. In the hands PIP, thumb interphalangeal (IP) and Distal Interphalangeal (DIP) joints are most involved in executing tasks, hence the use of gadgets such as adapted cutlery, can and bottle opener, zip puller and button hooks are recommended by occupational therapists to encourage joint protection and decrease dependency on others to help with such activities (i.e. the need for help and assistance), decrease pain and prevent joint damage. Therefore use of aids and environmental adaptations are viewed as enablers of function and independence, rather than a marker of disability. Nevertheless, the scoring system used within the HAQ [21, 22] increases with the use of special device by 1 point, if the patient ‘needs’ help from others to do an activity by 2 points and if the patient usually needs both a special device and help from another person by 3 points [54]. Moreover, permanent adaptations of the person’s environment (e.g. changing faucets in the bedroom or kitchen, using Velcro closures on clothing) are also counted as aids and devices [54]. This means, even if the responder has chosen to appraise their ability to do an activity as “without any difficulty” [scored 0] due to their use of aids and adaptations to enable function, their disability score will increase when they disclose the use of aids to help their functioning, unlike how the MAP-Hand is scored.

Another comparative scale that is commonly used in clinical and research practice and recently culturally validated for use in the UK is DASH [4]. DASH scores were also highly correlated with the MAP-Hand scores in this study (Table 6). DASH is a considerably larger, comprehensive scale of upper limb function, which also includes optional modules for assessing upper limb performance at work (WORKDASH) and measuring abilities and symptoms of athletes and performing artists (SPAMDASH). The optional modules are scored separately [4]. As the MAP-Hand, the DASH scores are calculated based on the responder’s ability, regardless of how they do the task. However, unlike the MAP-Hand the DASH includes items measuring both activity limitation (person-level) and participation restriction (societal-level) and as well as the activities performed using hands, the arm and shoulder function is also measured. Although DASH is a comprehensive assessment including 30 items, QUICKDASH is available as a shorter version and consists of 11 items (6 daily activity ability; two symptoms (pain and tingling); and three participation) [4, 55]. Therefore, although the MAP-Hand and the DASH questionnaires appear to measure the same construct at a first glance, their remits differ at conceptual and measurement level and clinicians and researchers should take these differences into consideration if they are having to choose the use of one measure over the other.

**Table 6:** Internal consistency and test-retest reliability of the British English MAP-Hand [Classical Testing]

	Cronbach’s alpha	n for test-retest	Test 1 score Mean [SD]	Test 2 score Mean [SD]	ICC(2,1) (95% CI)
MAP-Hand	0.96	273	17.61 [11.65]	17.08 [11.53]	0.96 (0.94,0.97)

### Statistical analysis

In this study the unidimensionality of the MAP-Hand was challenged by the CFA, but supported by the Rasch analysis. In both cases, substantial adjustments had to be made to accommodate the local dependency of the item set. The clusters of locally dependent items made clinical sense, grouping items with similar functional requirements, such as opening jars or cans.

Differential item function may also have contributed to the disturbance of dimensionality. The presence of DIF is not uncommon in health status measures of functioning [56]. The fact that, at any level of hand function males had more difficulty tying shoelaces may simply reflect that women are less likely to wear shoes with laces. Also that at any level of hand function, women have more problem opening jars may simply be a function of men having stronger grip. However at the scale level, the item DIF was cancelled out and, so as long as all 18 items are administered, the total score should remain unaffected.

In the analysis of the local independence, assumption took prominence, which was not reported in the original validation. This can inflate reliability, although in this case only marginally, and the level of reliability remained high, consistent with individual use. By using a testlet solution to absorb the local dependency, a satisfactory fit was achieved. The metric conversion that follows good fit will allow for an appropriate calculation of change scores, as well as aspects such as the minimal important difference, the calculations of which are invalid on ordinal scales [57].

### Limitations

We only tested the British MAP-Hand in people with RA. Further testing of the British MAP-Hand in other conditions is needed to ensure the scale has validity and reliability for use in these conditions. In addition, further studies should consider longitudinal design with multiple follow-up points to test the British English MAP-Hand’s ability to detect change in hand function over time (i.e. Responsiveness).

## Conclusions

The British English version of the MAP-Hand has been linguistically and culturally validated, and found to be a valid and reliable measure of hand function for people with RA in the UK. The British English MAP-Hand meets the COSMIN standards for evaluating the quality of PROM items [16] and can be used in both clinical practice and research.

## Additional files

Additional file 1: The Original MAP-Hand Assessment of hand function in activity performance. (DOCX 18 kb)

Additional file 2: British Measure of Activity Performance in the Hand [MAP-Hand]. (DOCX 18 kb)

## Abbreviations

CFA: Confirmatory Factor Analysis
CFI: Comparative Fit Index
DASH: Disabilities of the Arm Shoulder Hand
DIF: Differential Item Functioning
DIP: Distal Interphalangeal
DMARDs: Disease Modifying Anti-Rheumatic Drugs
HAQ: Health Assessment Questionnaire
ICC: Intra-Class Correlations
MAP-Hand: The Measure of Activity Performance of the Hand
MCP: Metacarpophalangeal
MDC^95^: Minimal Detectable Difference
NHS: National Health Service
NICE: The National Institute for Clinical Excellence
NRS: Numeric Rating Scales
PIP: Proximal Interphalangeal
PROMS: Patient Reported Outcome Measures
PSI: Person Separation Index
RA: Rheumatoid Arthritis
SD: Standard Deviation
SEM: Standard Error of Measurement
SF-36_v2_: Short-Form 36
TLI: Tucker Lewis Index
UK: United Kingdom
ULHAQ: Upper Limb HAQ
