# Supplementary material for: Measure of activity performance of the hand (MAP-Hand) questionnaire: linguistic validation, cultural adaptation and psychometric testing in people with rheumatoid arthritis in the UK
Source: BMC Musculoskelet Disord. 2018 Jul 31;19:275. doi: 10.1186/s12891-018-2177-5 (PMC6069818; doi:10.1186/s12891-018-2177-5)
Supplement: Supplementary file 1 — The Original MAP-Hand Assessment of hand function in activity performance. (DOCX 18 kb) [file 12891_2018_2177_MOESM1_ESM.docx]

# Additional file 1. The Original MAP-Hand Assessment of hand function in activity performance

# Please cross off the answer that best describes your ability to perform the activities the last time you performed them. If you used a technical aid, please assess your performance as it was with use of the technical aid.

|  | **No difficulty** | **Some difficulty** | **Great difficulty** | **Not able to do** |
| --- | --- | --- | --- | --- |

| 1. Buttoning buttons | 🞏 | 🞏 | 🞏 | 🞏 |
| --- | --- | --- | --- | --- |
| 1. Putting on socks or tights | 🞏 | 🞏 | 🞏 | 🞏 |
| 1. Tying shoelaces | 🞏 | 🞏 | 🞏 | 🞏 |
| 1. Squeezing out of tubes (e.g. toothpaste) | 🞏 | 🞏 | 🞏 | 🞏 |
| 1. Brushing teeth | 🞏 | 🞏 | 🞏 | 🞏 |
| 1. Wiping yourself after using the toilet | 🞏 | 🞏 | 🞏 | 🞏 |
| 1. Opening bottle screw tops | 🞏 | 🞏 | 🞏 | 🞏 |
| 1. Opening hermetic cans | 🞏 | 🞏 | 🞏 | 🞏 |
| 1. Opening jam jars | 🞏 | 🞏 | 🞏 | 🞏 |
| 1. Slicing bread using a knife | 🞏 | 🞏 | 🞏 | 🞏 |
| 1. Peeling raw vegetables | 🞏 | 🞏 | 🞏 | 🞏 |
| 1. Stirring food in a pan | 🞏 | 🞏 | 🞏 | 🞏 |
| 1. Wringing out cloths | 🞏 | 🞏 | 🞏 | 🞏 |
| 1. Carrying shopping bags | 🞏 | 🞏 | 🞏 | 🞏 |
| 1. Writing by hand | 🞏 | 🞏 | 🞏 | 🞏 |
| 1. Typing on a computer | 🞏 | 🞏 | 🞏 | 🞏 |
| 1. Pushing with hands when getting up from a chair | 🞏 | 🞏 | 🞏 | 🞏 |
| 1. Carrying heavy objects like suitcases and bags (over 5kg/ 10 lbs) | 🞏 | 🞏 | 🞏 | 🞏 |

*Originally developed in Norway, the MAP-Hand items are scored on a 4-item scale: No Difficulty [1]

Some Difficulty [2] Great Difficulty [3] Not Able to do [4] and summarised into a total score range [0 to 72].
