# Supplementary material for: Measure of activity performance of the hand (MAP-Hand) questionnaire: linguistic validation, cultural adaptation and psychometric testing in people with rheumatoid arthritis in the UK
Source: BMC Musculoskelet Disord. 2018 Jul 31;19:275. doi: 10.1186/s12891-018-2177-5 (PMC6069818; doi:10.1186/s12891-018-2177-5)
Supplement: Supplementary file 2 — British Measure of Activity Performance in the Hand [MAP-Hand]. (DOCX 18 kb) [file 12891_2018_2177_MOESM2_ESM.docx]

# Additional file 2. British Measure of Activity Performance in the Hand [MAP-Hand]

# This is an assessment of how you use your hands when doing everyday activities. Please tick the answer that best describes your ability to do the activities the last time you did them. If you use a gadget, please tick the answer that best describes your ability when using this.

|  | **No difficulty** | **Some difficulty** | **Great difficulty** | **Not able to do** |
| --- | --- | --- | --- | --- |

| 1. Buttoning buttons | 🞏 | 🞏 | 🞏 | 🞏 |
| --- | --- | --- | --- | --- |
| 1. Putting on socks or tights | 🞏 | 🞏 | 🞏 | 🞏 |
| 1. Tying shoelaces | 🞏 | 🞏 | 🞏 | 🞏 |
| 1. Squeezing out of tubes (e.g. toothpaste) | 🞏 | 🞏 | 🞏 | 🞏 |
| 1. Brushing teeth | 🞏 | 🞏 | 🞏 | 🞏 |
| 1. Wiping yourself after using the toilet | 🞏 | 🞏 | 🞏 | 🞏 |
| 1. Opening screw top bottles | 🞏 | 🞏 | 🞏 | 🞏 |
| 1. Opening cans (any type) | 🞏 | 🞏 | 🞏 | 🞏 |
| 1. Opening jam jars | 🞏 | 🞏 | 🞏 | 🞏 |
| 1. Slicing bread using a knife | 🞏 | 🞏 | 🞏 | 🞏 |
| 1. Peeling raw vegetables | 🞏 | 🞏 | 🞏 | 🞏 |
| 1. Stirring food in a pan | 🞏 | 🞏 | 🞏 | 🞏 |
| 1. Wringing out cloths | 🞏 | 🞏 | 🞏 | 🞏 |
| 1. Carrying shopping bags | 🞏 | 🞏 | 🞏 | 🞏 |
| 1. Writing by hand | 🞏 | 🞏 | 🞏 | 🞏 |
| 1. Typing on a computer | 🞏 | 🞏 | 🞏 | 🞏 |
| 1. Pushing with hands when getting up from a chair | 🞏 | 🞏 | 🞏 | 🞏 |
| 1. Carrying heavy objects like suitcases and bags (over 5kg/ 10 lbs) | 🞏 | 🞏 | 🞏 | 🞏 |

* The British MAP-Hand items are scored on a 4-item scale: No Difficulty [0] Some Difficulty [1] Great Difficulty [2] Not Able to do [3] and summarised into a total score range [0 to 54].
